# The interplay of actual and perceived motor competencies, physical activity and well-being: a child-centered approach

**DOI:** 10.1186/s12889-026-27094-w

**Published:** 2026-03-26

**Authors:** Kathrin Bretz, Johanna Kress, Christian Herrmann

**Affiliations:** Physical Education Research Group, Zurich University of Teacher Education, Zurich, 8090 Switzerland

**Keywords:** Motor development, Sports participation, Mental health, Child development, Physical education

## Abstract

**Background:**

Motor development models highlight the interplay between actual motor competence, perceived motor competence and physical activity in children. In studies, mental health parameters, such as well-being, are mostly considered as an outcome of PA or motor development, but they can also be the prerequisite for participation in PA. While variable centered studies have explored mostly correlations among these factors, there is a need to adopt person-centered approaches to identify distinct profiles based on these variables. Understanding these profiles can support targeted interventions to promote healthy motor development.

**Methods:**

We conducted a cross-sectional study involving *n* = 427 children (M = 7.78 years, SD = 0.70). Actual motor competence was assessed via motor competence test, perceived motor competence via self-perception and physical activity and well-being via parent questionnaires. Latent profile analysis was conducted to identify distinct profiles based on the characteristics of the investigated constructs.

**Results:**

Latent profile analysis revealed three distinct profiles. *Highly competent team players* (profile 1), who were characterized by high actual and perceived motor competencies in “object movement”, high participation in team sports and high well-being scores. *Competent self-movers* (profile 2), who exhibited good actual and perceived motor competencies, engaged primarily in individual sports and high well-being. *Low-participating overestimators* (profile 3), who demonstrated low actual motor competencies but high perceived motor competencies with an overestimation of their competencies, low involvement in organized sports and significant lower well-being compared to the other profiles.

**Conclusion:**

This study identifies distinct profiles in children’s motor development parameters, emphasizing the complex interplay between actual motor competencies, perceived motor competencies, physical activity and well-being. The presence of a profile with low actual motor competencies, physical activity and well-being indicates that a certain level of these factors may be necessary for active motor development. Tailored interventions should not only address physical activity or actual motor competencies but also encourage positive-realistic self-assessment and increased participation in physical activity, especially organized sports, to enhance both motor development and well-being.

**Supplementary Information:**

The online version contains supplementary material available at 10.1186/s12889-026-27094-w.

## Introduction

### Problem statement

In models of motor development, various factors have been identified that contribute to healthy child development, such as actual motor competence (AMC), perceived motor competence (PMC) and physical activity (PA). Stodden et al. [1] postulated a model for motor development in which the interplay and interactions of these factors are shown. In this model, the interplay of these factors can lead to a positive spiral of engagement, with higher PA, AMC, PMC and fitness, or a negative spiral of engagement, with lower PA, AMC, PMC and fitness, and a greater risk of obesity.

To date, these factors have often been examined via variable-centered approaches to identify correlations between each other and with other health-related factors, such as physical fitness or well-being [2–5]. By using a child-centered approach, some authors have focused on identifying different profiles of children by considering their AMC and PMC [6–8]. In these studies, different factors, such as sport participation or physical fitness, were associated with the identified profiles.

However, the aim of this study is to combine factors that contribute to healthy motor development (AMC, PMC, PA and well-being) via a child-centered approach to identify profiles with different characteristics and levels and investigate differences between the profiles regarding gender, age and BMI.

### Actual motor competence

Actual motor competence (AMC) refers to an individual’s level of proficiency across various motor tasks, encompassing the quality, coordination, and control involved in the motor outcomes [9, 10]. AMC can be divided into competence areas, e.g., *object control/object movement* and *locomotion/self-movement*. While object control encompasses forms of throwing, catching, or kicking a ball, locomotion refers to body control, such as running, balancing or jumping [11]. AMC can be accessed via product-oriented test instruments, e.g., the MOBAK-instruments, in which the quantitative assessments of the motor performance are examined (e.g., number of balls caught) [12] or process-oriented test instruments, such as the Test of Gross Motor Development which is used to examine qualitative movement aspects [13]. Due to the used test instruments and the MOBAK approach, “object movement” will be used simultaneously for object control and “self-movement” for locomotion.

According to the conceptual model of Hulteen and colleagues [14], AMC is the prerequisite for the development of sport-specific skills and for a physically active lifetime. This model emphasizes the importance of developing AMC in childhood, as a sufficient level of AMC is necessary to overcome the so called “proficiency barrier”, [14, 15] and to participate in the culture of sports and movement and develop sport-specific skills. Mastering such skills enables individuals to maintain an active lifestyle and further refine their physical fitness [16]. However, many environmental and biological factors impact the development of AMC. For example, biological factors such as sex and genetics, as well as environmental influences such as socialized gender roles, family support, peer interactions, and available opportunities for movement, play a role in shaping motor behavior and competence [17–19].

In recent years, various, mostly cross-sectional studies have been carried out, in which different correlations with AMC have been investigated. In different European countries, differences in the AMC levels have been reported between boys and girls. Boys have better AMC in “object movement” than girls, whereas girls perform better AMC in “self-movement” than boys do [12, 20, 21]. This difference may be due to gender preferences as boys participate more often in team sports, like soccer and other ball sports and thus improve their AMC in “object movement”, whereas more often engage in individual sports like dancing or gymnastics [22–24]. Differences were also found in age, whereby older children have a higher level of AMC than younger children do [25, 26]. Children who are more physically active have better AMC than children who are not [27]. With respect to different sport activities, children who participate in team sports (e.g., soccer, basketball) show better competencies in “object movement”, and children who participate in individual sports (e.g., track and field, gymnastics) show better competencies in “self-movement” [22, 28, 29].

According to Hulteen and colleagues [14], the stages of motor development are closely interlinked with both physical and psychological attributes, such as cardiorespiratory fitness or self-efficacy. In addition to AMC, perceived motor competence is recognized as an important factor influencing motor development.

### Perceived motor competencies

Perceived motor competence (PMC) refers to an individual’s belief or self-assessment of their ability to perform specific motor tasks [30]. Building on multidimensional, hierarchical models of self-concept that distinguish domain-specific self-perceptions (e.g., academic, social, physical), [31, 32] Estevan and Barnett [30] expanded the hierarchical model of the multidimensional structure of self-concept to incorporate PMC as a lower-order facet within the physical self-concept. In their adaption, PMC is subordinated to physical self-concept and includes perceived competence in stability skills, locomotor skills, object control skills and active play skills. PMC is seen as a crucial factor in the model of motor development, as it is both a mediator between physical activity and AMC and is directly associated with physical activity and AMC [33].

In early childhood, children are not yet able to reflect on their successes and failures, often overestimating their competence and skills. As their self-concept develops with increasing age and becomes more differentiated, they begin to recognize both successes and failures, engaging in peer comparison. This process should lead to the formation of a more realistic self-concept [34]. However, evidence suggests that even adolescents do not necessarily have aligned AMC and PMC [35].

As children with a low physical self-concept are less likely to engage in situations they perceive as challenging, they may miss crucial opportunities for motor development [1]. Consequently, PMC represents a particularly interesting construct for research, as it captures children’s subjective views of their own competence.

### Physical activity

Physical activity (PA) is crucial for the healthy growth and development of children and adolescents and plays a key role in preventing chronic diseases later in life and improving health-related outcomes [36–38]. A growing body of evidence highlights the relevance of PA as well as the widespread problem of insufficient PA across all age groups. In the global status report on PA, the World Health Organization (WHO) called low PA to be a global burden [39] and noted that 81% of boys and girls aged 11–17 years do not meet the WHO guidelines, recommending at least an average of 60 min per day of moderate- to vigorous-intensity PA [40]. The lack of PA is particularly alarming in children and adolescents, where insufficient PA can hinder motor development, limiting their ability to acquire essential movement skills needed for a healthy and active lifestyle. Conversely, insufficient AMC may lead to an inactive lifestyle [14]. The factors that influence participation in PA are complex and are attracting increasing interest in multiple scientific fields [41–43].

### Interplay of actual motor competencies, perceived motor competencies and physical activity

A sufficient level of AMC enables a child to engage in play and sports. The developmental model by Stodden et al. [1] proposes that the relationship between AMC and PA changes depending on the developmental stage of the child. In early childhood, children acquire AMC through movement, PA and different experiences in their environment, e.g., playgrounds, at home. In middle childhood, the relationship changes – the level of AMC enables a child to participate in sports and exercise. Cross-sectional studies revealed weak to moderate positive associations between AMC and PA in preschool children [44] and in children six years and older [45]. Field and colleagues [41] showed in their longitudinal study that children who overestimated their AMC increased their PA participation and AMC, whereas children who underestimated themselves had stable PA participation and little or no development in their AMC. Barnett and colleagues recently used pooled latent profile analysis to demonstrate that the high aligned (high AMC, high PMC) profile of AMC and PMC is associated with particularly high levels of physical activity, compared to the low aligned (low AMC, low PMC) profile [46]. In studies that considered the context of PA, e.g., individual or team sports, moderate associations between the type of sport and specific competence areas were found. Children who participate in team sports show higher levels of “object movement”, whereas children who are active in individual sports are better at “self-movement” [2, 29].

### Motor development and mental health

In recent years, especially during the COVID-19 pandemic, the well-being of children has gained increased interest [47], including in the field of motor development. Lima and colleagues [48] expanded Stodden et al.’s model [1] to include three groups of health parameters in the model, including (1) cognition and academic performance, (2) metabolic health and (3) mental health. In this model, PA, AMC, weight status and health-related fitness are expected to be directly associated with the three health parameters.

Mental health can be understood as a state of well-being in which people are able to handle everyday challenges, use their abilities, and engage in work, learning and social life [49]. Mental health can be operationalized in different ways, whereby mental health parameters can be clustered into well-being (e.g., global self-esteem, quality of life, well-being) and ill-being (e.g., anxiety, depression) [50–52]. Studies investigating mental health parameters revealed positive associations between PA and mental health parameters, whereby significant associations between PA and lower levels of psychological ill-being and greater psychological well-being were found [53–56]. Ibáñez-Román and colleagues [57] reported a dose‒response pattern in adolescence, whereby mental well-being positively predicted PA. The authors investigated the reverse pathway as well, whereby PA positively predicted mental well-being one year later. A review by White and colleagues [58] revealed evidence for affect, self-esteem and self-efficacy but also for social support, social connection and physical health. Moreover, during PA, people experience feelings of competence and self-efficacy, which can lead to greater mental health [58]. Organized sport is seen to have a positive effect on youth development by affecting physical (healthy lifestyle), psychological (building self-confidence) and social aspects (teamwork, social integration) [59]. A review by Boelens and colleagues [60] revealed a small positive impact of participating in organized sport activities on mental health parameters in children, whereas most of the studies included focused on mental health problems. Moreover, the authors hypothesized an indirect effect via PA. Bjørnarå and colleagues [61] reviewed reviews about organized sports participation and health in childhood and adolescence and reported moderate evidence between sport participation and reduced anxiety and depression. Low-to-moderate evidence was found for causal relationships between sport participation and psychological and social health. The evidence on the relationship between organized sports and mental health parameters remains inconclusive. This unclear, context-dependent relationship was also reported by Hoffmann et al. [62], who reported that participation in team sports was associated with better mental health parameters and that participation in individual sports was associated with lower mental health parameters.

With respect to AMC, there is less evidence for a positive relationship with mental health. Gu and colleagues [51] investigated the direct and indirect effects on mental health parameters through health-related physical fitness in adolescents. A positive significant relationship was found between AMC and mental health, whereby this effect was mediated through health-related physical fitness. The association between health-related quality of life (HRQoL) as a mental health outcome and AMC was investigated by Redondo-Tebar and colleagues [63], whereby a lower level of HRQoL was found in children with lower AMC levels, especially in children with developmental coordination disorder. The association between AMC and the physical dimension of HRQoL was mediated by cardiorespiratory fitness [5], which was also shown by Gu et al. [51]. Positive associations between general HRQoL and AMC were also evident in first- and second-grade children and between physical well-being and AMC in preschool children [64]. Wälti and colleagues [65] reported weak positive correlations between different subscales of HRQoL and children’s AMC in “self-movement”.

In the literature, global self-esteem is seen as an indicator of mental health or measured as a mental health parameter [52] but is also seen as a resilience factor for mental ill-being, such as anxiety or depression [66]. People with high self-esteem show better mental health parameters [67]. Visser and colleagues [68] investigated the impact of well-being and PMC on PA. PMC and self-esteem were both associated with PA at baseline and positively predicted PA one year later. Bardid and colleagues [69] investigated the levels of AMC and PMC via a person-centered approach and identified four groups of children. Children with high levels of both AMC and PMC and children with low levels of AMC and high levels of PMC presented higher levels of global self-worth than children with high levels of AMC and low levels of PMC and children with low levels of both AMC and PMC did, which highlights the importance of children’s PMC for their global self-worth. In general, there is still a research gap regarding the extent to which perceived competence influences global self-esteem. The Exercise and Self-Esteem Model [70] offers one possible answer, proposing that perceived improvements in competence affects self-esteem through bottom-up processes. This model has been empirically tested in several studies [71, 72]. Nevertheless, the social reference group is also crucial, as illustrated by the Big-Fish-Little-Pond effect [73, 74]. People’s self-perceptions vary depending on the reference group, such that comparison with a higher-performing group typically lowers, and comparison with a lower-performing group raises, self-evaluations of performance. The Big‑Fish‑Little‑Pond effect has been demonstrated for both academic [74] and physical self‑concept [75].

### Aim of the study

Owing to the small number of studies considering AMC, PMC, PA and mental health outcomes along with the use of mostly variable-centered approaches, we formulated two aims in the following study. The first aim of the current study was to identify different profiles of children based on their levels of AMC, PMC, PA and well-being as an outcome of mental health by using a person-centered approach. Although we used an exploratory approach for the analysis, we expected to find different profiles characterized by different combinations of levels of children’s AMC, PMC and PA. The second aim was to investigate whether the identified profiles differ according to BMI and gender, as these factors are seen as influencing factors in the context of motor development.

## Methods

The present study is based on the second measurement point (2023) of the longitudinal study “Development of basic motor competencies in children (EMOKK)” (2021–2025), in which additional instruments for the assessment of children’s PMC were used. The study was funded by the Swiss National Science Foundation (Project nr.: 200840). We assessed data from first and second grade primary school children in German-speaking Switzerland.

### Study design and participants

A total of 427 children (M = 7.78 years, SD = 0.70, 50.1% girls, 49.9% boys, 0.0% diverse) in the first and second grades of primary school participated in this study. The children were from different schools in the German-speaking cantons Zurich and Basel Land. The children were from 29 classes (average class size *n* = 15 children). Data were collected from three different sources: the AMC of the children were assessed by using a motor competence test (MOBAK instrument), the PMC was recorded by self-assessment of the children (SEMOK instrument) and the PA and well-being of the children were assessed via a parent questionnaire. These data sets were then merged. The AMC assessment, conducted during a PE lesson, involved *n* = 376 children, whereas the PMC data was gathered from *n* = 384 children in a regular classroom setting prior to the assessment. Additionally, the parents of *n* = 417 children completed the questionnaire at home. The study was conducted in accordance with the Declaration of Helsinki and approved by the Ethics Committee of the University of Zurich (Nr. 21.2.5, 19.12.2022). Informed consent was obtained from all parents of the participants, and participation was voluntary and could be canceled at any time.

### Measures

#### Basic motor competencies

To assess AMC, we used the MOBAK instrument for first- and second-grade primary school children (MOBAK-1-2), which operationalizes AMC as the level of basic motor competencies a child needs to participate in the culture of sport and movement [11, 76]. This instrument measures AMC in two competence areas: “object movement” and “self-movement”, each evaluated through four items (object movement: throwing, catching, bouncing, dribbling; self-movement: balancing, rolling, jumping, running). Each item is standardized with specific assessment criteria. During testing, each child had two attempts to complete the motor task, with no trial run. These attempts were scored on a dichotomous scale (0 = failed, 1 = successful), and the scores were summed to determine the final item score (0 points = no successful attempts, 1 point = one successful attempt, 2 points = two successful attempts). The tasks for throwing and catching were scored differently, with each child having six attempts. In these cases, 0–2 successful attempts were scored as 0 points, 3–4 as 1 point and 5–6 as 2 points. Each competency area had a maximum score of eight points. Data collection took place during a regular 45-minute PE class, during which the class was divided into small groups of three to four children, each guided through the eight test stations by trained testers. The testers provided a standardized explanation and a demonstration of each task. Additionally, children’s height (in cm) and weight (in kg) without shoes were measured during the MOBAK test to calculate their Body Mass Index (BMI).

#### Perceived basic motor competencies

PMC was assessed prior to AMC to ensure that PMC did not solely reflect the experience in the AMC test situation [77]. The children completed the SEMOK-1-2 questionnaire with eight illustrated tasks, which refer to the MOBAK-test items [28]. The assessment of PMC took place during the last 15 min of their regular classroom lesson, either just before or one or two days before the AMC assessment. Each child was given a double-sided sheet of paper listing the eight test items, with three horizontal circles displayed next to each task. Above these circles, the pictorial response options were illustrated: nodding, shrugging shoulders and shaking head. Once all the children had received their sheets, the study leader first explained the pictorial response options. Second, every task was described in detail, whereby the children had a brief moment to mark their response after each explanation [28].

For analysis, the pictorial response options were coded as follows: “positive”/nodding = 2 points, “neutral”/shrugging shoulders = 1 point, and “negative”/shaking head = 0 points. The points for each competence domain were then summed (0–2 points per item, max. 8 points per competence area). This alignment ensured that the tasks in the PMC instrument corresponded to those in the AMC instrument, allowing the scores for both instruments to be directly comparable.

#### Organized sports club participation

PA was operationalized via sports club participation. The children’s parents completed a questionnaire that included general information about their child and the child’s participation in organized sports club activities [78]. The question regarding sports club participation was asked in two steps. First, the parents were asked whether the child was a member of a sports club. If yes, they could tick a predefined sport on a list or provide an open answer. A maximum of two different sports club activities could be selected. For each sport, they could also tick the frequency with which the child attended the sport (0–7 times per week). The responses regarding the type and frequency of participation were categorized into team sports (e.g., basketball) or individual sports (e.g., track and field). These categories were then summed up to produce overall values for the frequency of participation in team sports and individual sports [78].

#### Health-related quality of life

Mental health parameters were assessed through health-related quality of life (HRQoL). A section of the parent questionnaire focused on assessing the children’s HRQoL. Therefore, the KIDSCREEN-27 questionnaire, which is an established and validated instrument for the assessment of HRQoL, that is available in multiple languages, was used [79]. The KIDSCREEN-27 included questions covering five areas of well-being, including “physical well-being” (4 items, e.g., Has your child felt fit and well? ), “psychological well-being” (7 items, e.g., Has your child been in a good mood? ), “autonomy and parent relation” (7 items, e.g., Has your child had enough time for him/herself? ), “social support and peers” (4 items, e.g., Has your child had fun with his/her friends? ) as well as “school environment” (4 items, e.g., Has your child been able to pay attention? ). The mean values for each individual subscale were calculated for further analysis. For our analysis, physical, psychological and social well-being were included.

### Statistical analysis

In the first step, we calculated descriptive data by using SPSS 29 [80]. Therefore, mean values (M), standard deviations (SD) and 95% confidence intervals (95%-CI) were calculated. The BMI was used in its raw form, without z-score standardization. Moreover, we calculated the estimation of children’s motor competencies, which was shown through the Delta (Δ) by calculating the difference between PMC and AMC in both competence areas. Differences between boys and girls were investigated by using t-tests. Bivariate Pearson-correlations between PA, AMC, PMC and well-being were carried out. Effect sizes were interpreted via Cohen’s [81] guidelines, with *r = *|0.10| as a small effect, *r = *|0.30| as a medium effect and *r = *|0.50| as a strong effect and *η*^*2*^ values of 0.01, 0.06 and 0.14 indicating small, medium and large effects, respectively. Cohen’s *d* was interpreted as *d *= 0.20 for a small effect, *d *= 0.50 for a medium effect and *d *= 0.80 for a large effect.

To consider a person-centered approach, latent profile analysis (LPA) was conducted via Mplus version 8 [82]. LPA is a statistical method used to identify distinct subgroups within a population that may share similar observable characteristics. The core assumption of LPA is that the membership in these unobserved profiles can be explained based on patterns of variables, e.g., out of observations, assessments or questionnaires. In this study, the aim was to identify children with different profiles based on their PA, AMC, PMC and well-being. We included nine variables in the LPA: the frequency of team sports (1), AMC “object movement” (2), PMC “object movement” (3), physical well-being (4), psychological well-being (5), social well-being (6), PMC “self-movement” (7), AMC “self-movement” (8) and the frequency of individual sports (9). Missing data were handled via full information maximum likelihood (FIML). We started with the estimation of one profile model and added profiles until the optimal number of latent profiles with the best statistical and theoretical solutions was found.

Based on the recommendations of Geiser [83] and Weller and colleagues [84], the following criteria were established for determining the optimal number of profiles:

- Model fit: The Bayesian information criterion (BIC), the Akaike information criterion (AIC) and the adjusted Bayesian information criterion (ABIC) indicate the fit of the model. Low values indicate better model fits [83]. The average latent posterior probabilities indicate the likelihood of an individual being assigned to a specific profile. Higher values, nearing 1, are preferable, with some researchers suggesting a cutoff of 0.80 as acceptable and values above 0.90 as ideal [84]. Entropy reflects how precisely the model distinguishes between classes, where a value close to 1 is optimal, and anything above 0.80 is considered acceptable [84].
- Interpretability: The number of profiles should be theoretically meaningful and interpretable regarding the research question [83, 84].
- Parsimony: A model with fewer profiles should be chosen to avoid overfitting but also ensure a good explanation of the data [83].
- Class size: The sizes of the latent profiles should be reasonable and not too small to represent distinct groups. Shanahan and colleagues [85] recommend groups not smaller than 5%.

However, the authors noted that theoretical interpretation is decisive for the selection of the optimal number of profiles, supported by the statistical model fits. After identifying the optimal number of latent classes, we calculated one-way ANOVAs as well as descriptive statistics with SPSS 29 to investigate the differences between the profiles regarding AMC, PMC, PA, well-being, gender, age and BMI [80].

## Results

First, descriptive results were calculated for the overall sample as well as separately for boys and girls (see Table 1). The findings revealed a significant difference between boys and girls in the competence area “object movement”, in which boys performed better (AMC: *p *< 0.001, *d *= 0.57) and rated their own competencies (PMC: *p *< 0.001, *d *= 0.99) higher than girls did. Girls showed better results in AMC “self-movement” (*p *= 0.011, *d *= -0.27), whereas their perceptions did not differ from those of the boys (*p *= 0.361, *d *= -0.09). These findings regarding the differences between boys and girls were as expected and aligned to previous studies [20, 86]. All children overestimated their competencies, whereby the children rated their competencies more accurately in “object movement” (Δ = 0.56) than in “self-movement” (Δ = 1.54). In both competence areas, the difference between PMC and AMC was greater in boys (Δ “object movement”: *p *= 0.042, *d *= 0.21; Δ “self-movement”: *p *= 0.021, *d *= 0.25). On average, children were active 0.56 days per week in team sports and 1.07 days per week in individual sports, whereas boys were more active in team sports (*p *< 0.001, *d *= 0.58) and girls were more active in individual sports (*p *= 0.001, *d *= -0.32). In physical, psychological and social well-being, no significant differences between boys’ and girls’ well-being were found.

As in previous studies, manifest positive associations between PA and both AMC and PMC were found, whereby children in team sports showed higher AMC (*r *= 0.202, *p *< .001) and PMC (*r *= 0.307, *p *< .001) in “object movement”, whereas children in individual sports showed higher values in AMC (*r *= 0.11, *p *< 0.05) and PMC (*r *= 0.15, *p *< 0.01) in “self-movement” (see Supplementary Table 1). Moreover, children with better AMC showed higher levels in PMC in both competence areas (“object movement”: *r *= 0.39, *p *< 0.001, “self-movement: *r *= 0.26, *p *< 0.001). Correlations with well-being were found for AMC “self-movement” and physical well-being (*r *= 0.11, *p *< 0.05) as well as social well-being (*r *= 0.13, *p *< 0.05). The frequency of team sports was also positively associated with children’s social well-being (*r *= 0.11, *p *< 0.05).

**Table 1:** Descriptive statistics of AMC and PMC, frequency of sport participation and different areas of wellbeing for the total sample and boys and girls separately

	Total		Boys		Girls		
	*N*	M [CI 95%]	*n*	M [CI 95%]	*n*	M [CI 95%]	d
AMC Object movement^a^	369	5.76 [5.60; 5.93]	179	6.23 [6.01; 6.45]	190	5.33 [5.09; 5.57]	0.57***
AMC Self-movement^a^	352	5.70 [5.52; 5.89]	174	5.46 [5.18; 5.74]	178	5.94 [5.69; 6.19]	− 0.27*
PMC Object movement^a^	384	6.32 [6.17; 6.46]	189	6.98 [6.81; 7.15]	195	5.67 [5.47; 5.87]	0.99***
PMC Self-movement^a^	359	7.23 [7.13; 7.33]	189	7.18 [7.02; 7.34]	195	7.27 [7.15; 7.39]	− 0.09
Δ Object movement^b^	359	0.56 [0.38; 0.74]	175	0.75 [0.52; 0.98]	184	0.38 [0.11; 0.65]	0.22*
Δ Self-movement^b^	343	1.54 [1.35; 1.72]	170	1.76 [1.48; 2.04]	173	1.32 [1.07; 1.56]	0.25*
Frequency team sports^c^	407	0.53 [0.44; 0.62]	206	0.79 [0.64; 0.94]	201	0.27 [0.18; 0.35]	0.58***
Frequency individual sports^c^	407	1.07 [0.96; 1.17]	206	0.90 [0.77; 1.03]	201	1.24 [1.08; 1.40]	− 0.32**
Physical well-being^d^	417	4.39 [4.34; 4.44]	211	4.39 [4.32; 4.47]	206	4.39 [4.33; 4.46]	0.002
Psychological well-being^d^	416	4.31 [4.28; 4.35]	210	4.30 [4.24; 4.35]	206	4.33 [4.28; 4.38]	− 0.08
Social well-being^d^	414	4.13 [4.08; 4.19]	209	4.09 [4.01; 4.17]	205	4.18 [4.11; 4.25]	− 0.17

***p < 0.001, **p < 0.01, *p < 0.05; ^a^Range: 0–8, ^b^Range: -8–8, ^c^Days per week, ^d^Range: 1–5

### Latent profile analysis

The aim of the LPA was to identify groups of children with similar characteristics. The profiles were determined through a multistage process, with the model fits for the different solutions presented in Table 2. The results show that the values of the AIC, BIC and ABIC decreased with increasing profiles. As the model fits AIC, BIC, and ABIC did not show a statistically clear optimal number of latent profiles, we considered the sample proportions per profile, the classification accuracy and a theoretical explanation to determine the number of identified profiles. According to Nylund-Gibson and Choi [87], there is no single agreed-upon fit index for enumerating profiles but a set of different fit indices. The three-profile solution showed an acceptable entropy of 0.883, good classification accuracy (> 0.80), and the smallest class contained more than 5% of the sample. Despite the selection of numerous random starts, the optimal log-likelihood could not be replicated for the four-profile solution, and a small sample proportion of some profiles appeared (1% of the sample). Owing to these reasons and the theoretical interpretation, we decided on the three-profile solution.

**Table 2:** Model fit indices for latent profile analysis

Number of profiles	AIC	BIC	ABIC	Entropy	VLMR	aLMR	BLRT
1 Profile	9309.675	9382.697	9325.576	-			
2 Profiles	9089.225	9202.815	9113.961	0.942	0.279	0.282	< 0.001
3 Profiles	**8932.421**	**9086.579**	**8965.991**	**0.883**	**0.229**	**0.234**	**< 0.001**
4 Profiles	8702.949	8897.674	8745.352	0.973	0.732	0.734	< 0.001

The bold values indicate the selected three-profile solution

**Fig. 1:**
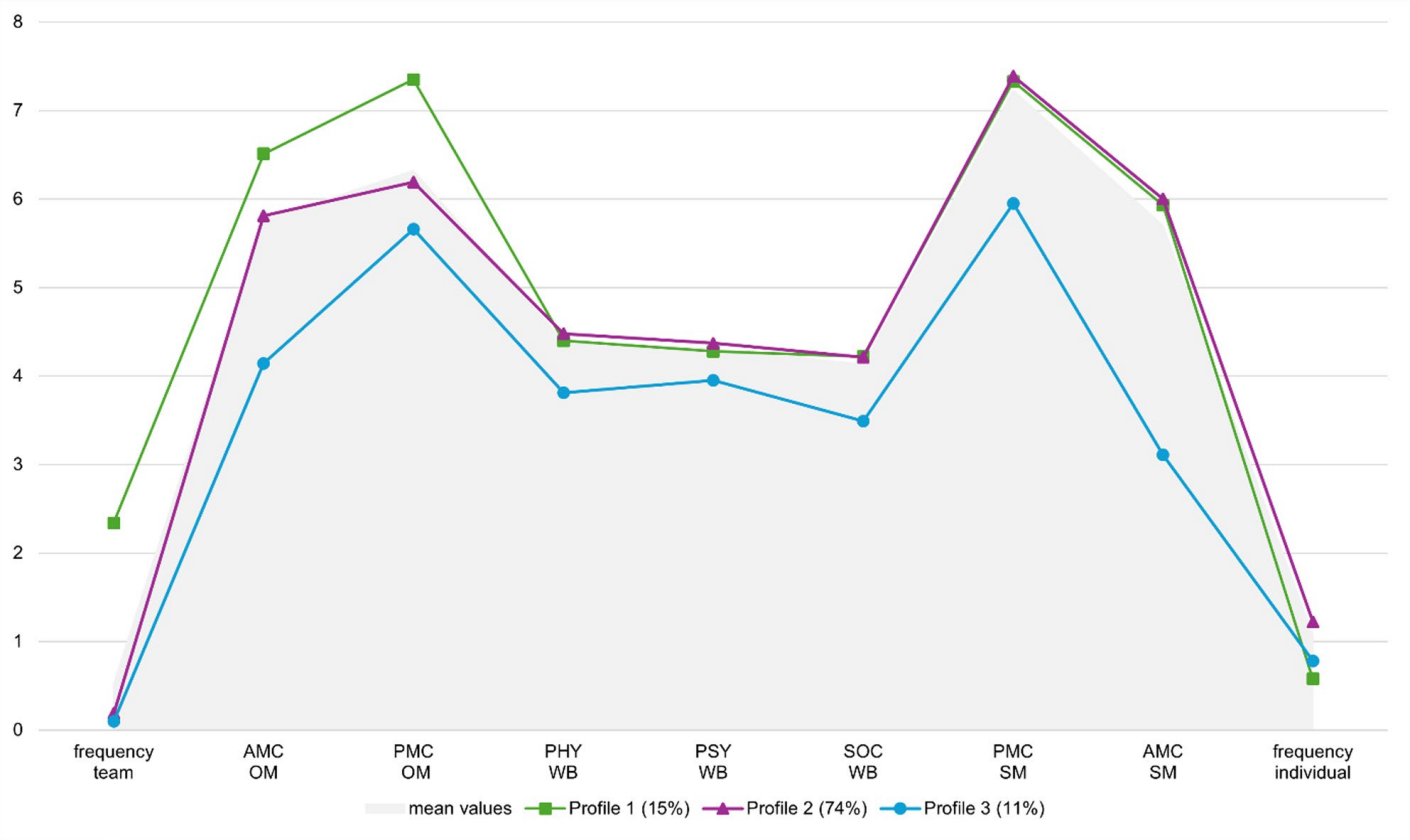
Latent profile results: mean values for the frequency of team sports (frequency team; 0–7 days per week) and individual sports (frequency individual; 0–7 days), actual motor competence (AMC; 0–8 points) and perceived motor competence (PMC; 0–8 points) motor competence in object movement (OM) and self-movement (SM), physical (PHY; 1–5 points), psychological (PSY; 1–5 points) and social (SOC; 1–5 points) well-being (WB) profiles. The green line represents Profile 1, the lilac line represents Profile 2, the blue line represents Profile 3, and the gray line represents the mean value of the total sample

Figure 1 shows the graphic representation of the three-profile model, whereby the x-axis lists the nine variables we included in the profile analysis. The y-axis shows the level of the individual variables (AMC and PMC: 0–8 points; frequency of individual/team sports: 0–7 days per week; physical, psychological and social well-being: 1–5 points).

#### Profile 1 (green)

comprised 15% of the children and was characterized by high sport participation in team sports and low participation in individual sports (*Highly competent team players*). This group exhibited high levels of AMC in both “object movement” and “self-movement” as well as high levels in PMC “object movement” and “self-movement”. Additionally, profile 1 showed high level of physical, psychological and social well-being.

#### Profile 2 (purple)

represented the majority of the sample with 74%. The children in this class showed little engagement in team sports but participated in individual sports approximately 1.2 days per week (*Competent self-movers*). Their AMC and PMC scores for “object movement” were slightly lower than those in profile 1, but their scores for both AMC and PMC “self-movement” were similar to those in profile 1. The well-being of children in profile 2 was comparable to the well-being of children in profile 1.

#### Profile 3 (blue)

accounted for 11% of the children in the sample and was characterized by low participation in both team and individual sports (*Low-participating overestimators*). This group displayed lower AMC scores for “object movement” and low values for AMC “self-movement” but high values for PMC “self-movement”. Profile 3 showed the greatest difference between PMC and AMC in both competence areas, which means that there was a discrepancy between their own perception of their competence and their AMC. Moreover, the children in profile 3 had significantly lower levels of physical, psychological and social well-being than the children in profiles 1 and 2 did.

As shown in Fig. 1, the children in profile 3 (*Low-participating overestimators*) presented lower values for all the investigated constructs than the *highly competent team players* (Profile 1) and *competent self-movers* (Profile 2) as well as the total sample did. Children in profile 1 (green) and profile 2 (purple) attained similar levels of well-being, AMC and PMC in “self-movement”. After implementing the profile membership into the dataset, we calculated the mean differences (MD) in PA, AMC, PMC and well-being between the different profiles via ANOVA. Profile 1 and profile 2 differed in the competence area “object movement” in both AMC (MD = 0.70, *p *< 0.002) and PMC (MD = 1.16, *p *< 0.001) as well as in their sport participation: the children in profile 1 were more active in team sports (MD = 2.15, *p *< 0.001), whereas the children in profile 2 were more active in individual sports (MD = 0.63, *p *< 0.001).

As shown in Fig. 1 and Table 3, the *low-participating overestimators* in profile 3 had the lowest levels of physical, psychological and social well-being and had significantly lower AMC in both competence areas. The *low-participating overestimators* also had significantly lower levels in their PMC but the highest deviations between their AMC and PMC (Δ object movement = 1.34, Δ self-movement = 2.86), which means that they overestimated themselves more strongly than the *highly competent team player* and the *competent self-movers* did, especially in the competence area “self-movement”.

**Table 3:** Mean values and 95% confidence intervals (95% CI) of the variables by profile

	Total (*n* = 427)	Profile 1 (*n* = 66)	Profile 2 (*n* = 316)	Profile 3 (*n* = 45)	ANOVA between profile 1, profile 2 and profile 3		
	M [CI 95%]	M [CI 95%]	M [CI 95%]	M [CI 95%]	F	*p*	η^2^
AMC Object movement^a^	**5.76 ** **[5.60; 5.93]**	6.51 [6.20; 6.82]	5.81 [5.63; 5.99]	4.14 [3.45; 4.84]	(2, 366) = 26.439	< 0.001	0.126
AMC Self-movement^a^	**5.70 ** **[5.52; 5.89]**	5.93 [5.45; 6.41]	6.00 [5.82; 6.18]	3.11 [2.56; 3.67]	(2, 349) = 53.763	< 0.001	0.236
PMC Object movement^a^	**6.32 ** **[6.17; 6.46]**	7.35 [7.12; 7.58]	6.19 [6.02; 6.36]	5.66 [5.23; 6.09]	(2, 381) = 22.444	< 0.001	0.105
PMC Self-movement^a^	**7.23 ** **[7.13; 7.33]**	7.33 [7.10; 7.57]	7.39 [7.30; 7.47]	5.95 [5.47; 6.43]	(2, 381) = 48.068	< 0.001	0.201
Δ Object movement^b^	**0.56 ** **[0.38; 0.74]**	0.84 [0.48; 1.20]	0.40 [0.19; 0.60]	1.34 [0.57; 2.11]	(2, 356) = 5.754	0.003	0.031
Δ Self-movement^b^	**1.54 ** **[1.35; 1.72]**	1.40 [0.88; 1.91]	1.38 [1.19; 1.58]	2.86 [2.06; 3.65]	(2, 340) = 11.475	< 0.001	0.063
Frequency team sport^c^	**0.53 ** **[0.44; 0.62]**	2.34 [2.13; 2.55]	0.19 [0.15; 0.24]	0.10 [0.01; 0.19]	(2, 404) = 535.812	< 0.001	0.726
Frequency individual sport^c^	**1.07 ** **[0.96; 1.17]**	0.58 [0.41; 0.76]	1.22 [1.09; 1.35]	0.78 [0.53; 1.04]	(2, 404) = 11.707	< 0.001	0.056
Physical well-being^d^	**4.39 [4.34; 4.44]**	4.40 [4.25; 4.54]	4.48 [4.43; 4.52]	3.81 [3.63; 4.00]	(2, 414) = 38.938	< 0.001	0.158
Psychological well-being^d^	**4.31 [4.28; 4.35]**	4.28 [4.17; 4.40]	4.37 [4.34; 4.41]	3.95 [3.81; 4.08]	(2, 413) = 26.736	< 0.001	0.115
Social well-being^d^	**4.13 [4.08; 4.19]**	4.22 [4.10; 4.34]	4.21 [4.15; 4.26]	3.49 [3.25; 3.74]	(2, 411) = 37.538	< 0.001	0.154
BMI	**16.04 [15.79; 16.29]**	16.27 [15.67; 16.87]	15.85 [15.58; 16.12]	17.07 [15.88; 18.26]	(2, 374) = 4.381	0.013	0.023
Age (in years)	**7.78 [7.71; 7.84]**	7.95 [7.80; 8.10]	7.79 [7.71; 7.86]	7.47 [7.22; 7.72]	(2, 421) = 6.519	0.002	0.030

		**Profile 1**	**Profile 2**	**Profile 3**	**χ** ^**2**^	***p***	
Gender (girls)	**50.1%**	18.2%	58.5%	37.8%	(2) = 38.642	< 0.001	
Gender (boys)	**49.9%**	81.8%	41.5%	62.2%			

Values in bold from the total sample. ^a^Range: 0–8, ^b^Range: -8–8, ^c^Days per week, ^d^Range: 1–5

### Differences in gender, age and BMI between the profiles

In addition to the identification of the latent profiles and the differences between the profiles, we also calculated differences regarding gender, age and BMI. Profile 1 contained 81.8% boys, whereas profile 2 contained 41.9% boys and 58.1% girls, which was almost balanced between genders. Profile 3 included more boys than girls, with 62.2% boys (Table 3). With respect to age, a difference with a medium effect size appeared between the profiles (*η*^*2*^ = 0.030), whereby only nonoverlapping 95% confidence intervals between children in profile 1 and profile 3 were observed. The children in profile 3 were slightly younger than the children in profile 1 were, but in comparison with the mean age of the total sample, no age difference was found. With respect to BMI, small differences were found between the profiles (*η*^*2*^ *=* 0.023), whereby children from profile 3 had a slightly higher BMI than did children from the other two profiles but with overlapping CIs.

## Discussion

This study aimed to identify distinct profiles (research question 1) of children based on their actual motor competence (AMC), perceived motor competence (PMC), physical activity (PA) as well as physical, psychological and social well-being and to examine whether these profiles differed in terms of gender, age and BMI (research question 2). The results reveal three distinct groups of children, each characterized by different patterns of AMC and PMC, sports participation, and well-being. By taking a person-centered approach, our study provides a holistic perspective on the interplay of these variables, expanding upon previous research that focused primarily on variable-centered analyses.

### Interpretation of the profiles

We identified three groups of children whose characteristics significantly differed in all investigated variables:

#### Profile 1: *Highly competent team players* (15%)

Children in this profile exhibited high levels of AMC and PMC in “self-movement”, very high levels of AMC and PMC in “object movement” and were actively engaged in team sports. Their well-being scores were within the normative range [88]. These findings align with those of previous studies suggesting that participation in organized sports fosters AMC, enhances PMC, and contributes positively to well-being. This profile had the highest proportion of boys (81.8%), which is consistent with the literature indicating that boys are more likely to engage in team sports and demonstrate higher AMC and PMC in object movement [22, 23, 89].

#### Profile 2: *Competent self-movers (*74%)

The majority of the children fell into this category, exhibiting high AMC and PMC in “self-movement” but lower scores in “object movement” than children in profile 1 did. These children were more active in individual sports than in team sports. Their well-being was within the normative range [88] and similar to the well-being of children in profile 1, suggesting that a certain level of competence – regardless of the type of sports the children were doing – may be sufficient to maintain well-being. The gender distribution in this profile was relatively balanced (boys: 41.5%, girls: 58.5%).

Children in profile 1 and profile 2 had a positive self-perception of their motor competence in “object movement” (profile 1: Δ = 0.84; profile 2: Δ = 0.40) and “self-movement” (profile 1: Δ = 1.40; profile 2: Δ = 1.38), with a small overestimation of their motor competencies, what is in alignment with the literature as young children generally overestimate their competencies [34].

#### Profile 3: *Low-participating overestimators* (11%)

Children in this profile had low AMC in both “object movement” and “self-movement” but exhibited high PMC, indicating an overestimation of their competencies. Their participation in organized sports was low, and their well-being scores were significantly lower than those of the children in profiles 1 and 2.

While children in profiles 1 and 2 had positive-realistic self-perceptions, the discrepancy between AMC and PMC in profile 3 indicates a high overestimation of their motor competencies in both “object movement” (Δ = 1.34) and “self-movement” (Δ = 2.86). Other authors using person-centered approaches also identified profiles of children with low AMC and high PMC [2, 90]. While a slight overestimation of competence could be beneficial for motivation and engagement [7, 34], excessive overestimation could lead to repeated failure experiences and even increased risk of injury owing to an inability to accurately judge one’s own capabilities.

### Gender, age and BMI differences across profiles

The gender distribution varied across profiles, with more boys represented in profile 1 and profile 3, whereas profile 2 showed a more balanced gender ratio. These findings support previous research indicating that boys are more likely to engage in structured team sports (profile 1), which contributes to greater AMC, whereas girls are more likely to participate in individual or self-directed activities (profile 2) [23–25]. The overrepresentation of boys in profile 3 may suggest that boys’ well-being could be more affected by low AMC and low PA participation, particularly in terms of social well-being. In older children and adolescents PA and AMC tend to be more important for males than for females [91]. If boys become aware of their deficits in AMC and realize that these limitations prevent them from fully joining in and participating in activities, this awareness may negatively affect their well-being (e.g., boys in profile 3).

The age differences were very low, although ANOVA revealed a significant difference between profile 1 and profile 3, whereby the children in profile 3 were younger than children in profile 1. Given that all the children included in this study were in first or second grade, we expected small age differences. With respect to BMI, children in profile 3 had significantly higher BMI values than those in the other two profiles did. This aligns with previous studies suggesting that low AMC, low PA, and higher BMI are interconnected [1].

### The complex relationships among motor competence, well-being and physical activity

The results show that there appear to be interactions between the variables that cannot be revealed in bivariate correlation analyses. The children in profile 3 presented lower values for all the variables than their peers did except PMC. Hulteen and colleagues [14] developed a conceptual model for the development of foundational movement skills for PA across the lifespan, indicating a “proficiency barrier”, which can be overcome with sufficient AMC to develop further specialized movement skills. Considering the idea of a proficiency barrier, it could be assumed that insufficient AMC is not the only barrier that children need to overcome in their motor development process: they also might need a positive-realistic estimation of their competencies to continue practicing things they cannot do thus far but also estimate their own physical limitations and compare their AMC with those of their peers. Moreover, children need opportunities for PA and interactions with peers, such as organized sport activities, as well as safe movement areas, such as playgrounds outside or safe routes to school [42, 92]. As mental health is seen as both an outcome and a predictor of PA [93], mental health could also be seen as a barrier for motor development, indicating that children can participate in PA when they feel socially integrated (social well-being), physically able (physical well-being) and be mental well and self-effective (psychological well-being). Schools could provide targeted PA opportunities in which children feel socially included, regardless of their competence level. This might include activities tailored to different ability levels, mixed-competence groups and a shift in extracurricular programs from purely competitive formats towards more cooperative structures that emphasize collaboration. As teacher support is also important for children’s PA, teachers should be aware of their key role, especially in PE [42].

In summary, two of the profiles we identified (profiles 1 and 2) were characterized by good AMC and PMC, regular sport participation and good well-being values. These relationships and the context-specific effects of PA on competence areas have been investigated in previous studies [22, 29, 94]. Moreover, children who were active in organized sports activities outperformed children who were not [95] and showed better coordination levels, especially when they consistently participated [96]. In contrast, children in profile 3 were rarely active in organized sports, had low AMC, whereby they overestimated themselves strongly and showed significantly lower well-being values than children in the other two profiles did. Other authors using person centered approaches found that children in profiles characterized by low AMC and low PMC not only showed less PA than those in high AMC-high PMC profiles [46, 97], but also reported lower motivation and reduced enjoyment of PE [2]. Since PMC is shaped by social comparison, such as in sports clubs, the strong overestimation observed in profile 3 may result from a lack of opportunities for comparison due to their limited participation.

By moving away from traditional variable-centered approaches, this study provides a holistic view of how these factors interact within individual children, potentially revealing unique profiles that may be obscured in aggregate analyses. By considering the directions of effects of the model proposed by Stodden et al. [1] and the positive and negative spiral of development, we can conclude that the interplay of strongly pronounced factors related to motor development leads to positive development and that the interplay of weakly pronounced factors leads to negative development in which children are “at risk” for motor development. The findings support this assumption, since it is not clear which of the investigated variables is discriminatory for the low level of mental health in profile 3. However, it is conceivable that it is not just one variable that is crucial for the low level of well-being, but the interaction of low levels of these variables. Moreover, children in profile 3 had significantly higher BMI scores, supporting the developmental model [1], which posits that children with low AMC, PMC and PA are at greater risk for overweight and obesity. The low level of sports participation in this profile may be a key contributing factor. Importantly, their lack of participation in organized sports may also limit their opportunities for social comparison, which is crucial for developing a realistic self-concept [98]. This raises concerns about the role of peer interactions in shaping self-perceptions and how the absence of structured PA environments might hinder a more accurate self-assessment of competence. While children strongly overestimate themselves, their well-being values are lower than those of the other profiles. One possible explanation is that PMC alone is not sufficient – children may also require positive movement experiences and mastery to build well-being. Lower participation in organized sports could also be associated with fewer social ties and friendships (e.g., less time with peers, fewer shared group activities). Our data do not include measures of social relationships, so we cannot test this assumption, but there is evidence that children with higher AMC are socially better integrated and more popular than children with low AMC [64, 99, 100]. Therefore, this is mentioned only as a potential explanation that should be examined in future studies.

To our knowledge, our study is the first to integrate AMC, PMC, PA and mental health into a person-centered approach by considering these variables. Another strength of our study is that we assessed AMC (motor competence test), PMC (self-assessment), PA and well-being (parent questionnaire) from three different perspectives. Moreover, we assessed the content in which children move by asking whether they are in a sport club, and which sports they participate in.

### Limitations

While this study offers valuable insights, several limitations should be acknowledged. First, although we considered different perspectives of motor tests, self-assessments and parent questionnaires, we did not collect self-reported well-being or PA data from the children because of time constraints and limited resources in the EMOKK-study. Future studies should include children’s self-reported well-being and physical activity to strengthen the findings, by using child appropriate instruments [101, 102]. Second, we did not include other PA behaviors, such as outdoor play and active commuting, which could influence motor competence development or well-being. Previous studies have shown that children who participate in organized sports clubs are also more physically active outside of structured activities, indicating the relevance of organized PA activities [103, 104]. As other PA activities, especially playing outside in nature, are positively related to children’s mental health parameters [105], the context, in which children move should be considered in future studies. Third, we only included children from first and second grade of primary school in our study. Therefore, the findings cannot be generalized to younger or older children. Fourth, our study design was cross-sectional, whereby we cannot investigate developmental changes over time. Longitudinal studies are needed to investigate whether children remain in their respective profiles or transition between them during development.

### Conclusion

Our findings highlight the importance of providing children with opportunities to engage in structured PA and developing realistic perceptions of their motor competence. It is important to provide children with activities or learning environments that are supportive and that children feel a sense of belonging. Furthermore, children can be encouraged to participate in organized sports. Interventions should be tailored to address deficits not only in PA or AMC but also in self-assessment to promote a positive-realistic self-concept, particularly for children in profile 3, who may be at risk for long-term disengagement from PA and low well-being. Additionally, research on PA and well-being should consider not only the quantity but also the context in which PA occurs, such as organized sports, peer interactions and free play.

It is crucial to empirically test models that integrate mental health parameters [48] and consider mental health not only as an outcome but also as a predictor of healthy child development. More comprehensive approaches with child-centered approaches are needed to understand and support children’s physical and emotional development by addressing multiple interacting influences on motor development and identify children at risk for motor development but also low well-being.

## Supplementary Information

Supplementary Material 1.

## Data Availability

The materials and datasets generated and analyzed during the current study are available from the corresponding author on reasonable request.

## Abbreviations

AMC: Actual Motor Competence
PMC: Perceived Motor Competence
PA: Physical Activity
BMI: Body-Mass-Index
PE: Physical Education
WHO: World Health Organization
HRQoL: Health-Related Quality of Life
