# Supplementary material for: The interplay of actual and perceived motor competencies, physical activity and well-being: a child-centered approach
Source: BMC Public Health. 2026 Mar 26;26:1464. doi: 10.1186/s12889-026-27094-w (PMC13141401; doi:10.1186/s12889-026-27094-w)
Supplement: Supplementary file 1 — Supplementary Material 1. [file 12889_2026_27094_MOESM1_ESM.docx]

Supplementary Table 1: Manifest bivariate Pearson correlations between the investigated constructs.

|  | (2) | (3) | (4) | (5) | (6) | (7) | (8) | (9) |
| --- | --- | --- | --- | --- | --- | --- | --- | --- |
| (1) AMC “Object movement” | **.302***** | **.389***** | **.125*** | **.202***** | .012 | .060 | .004 | .011 |
| (2) AMC “Self-movement” |  | -.004 | **.255***** | .043 | **.112*** | **.105*** | .085 | **.131*** |
| (3) PMC “Object movement” |  |  | **.237***** | **.307***** | -.067 | .056 | -.011 | .043 |
| (4) PMC “Self-movement” |  |  |  | .058 | **.146**** | .057 | .050 | .090 |
| (5) Frequency of team sports |  |  |  |  | **-.214***** | .093 | .069 | **.113*** |
| (6) Frequency of individual sports |  |  |  |  |  | .052 | .070 | .038 |
| (7) Physical well-being |  |  |  |  |  |  | **.482***** | **.409***** |
| (8) Psycho-logical well-being |  |  |  |  |  |  |  | **.459***** |
| (9) Social well-being |  |  |  |  |  |  |  |  |

Note: significant correlations in bold, ****p*<.001, ***p*<.01, **p*<.05
